# Dietary Resistant Starch From Potato Regulates Bone Mass by Modulating Gut Microbiota and Concomitant Short-Chain Fatty Acids Production in Meat Ducks

**DOI:** 10.3389/fnut.2022.860086

**Published:** 2022-03-17

**Authors:** Huaiyong Zhang, Simeng Qin, Yao Zhu, Xiangli Zhang, Pengfei Du, Yanqun Huang, Joris Michiels, Quifeng Zeng, Wen Chen

**Affiliations:** ^1^Key Laboratory of Animal Biochemistry and Nutrition, College of Animal Science and Technology, Ministry of Agriculture, Henan Agricultural University, Zhengzhou, China; ^2^Laboratory for Animal Nutrition and Animal Product Quality, Department of Animal Sciences and Aquatic Ecology, Ghent University, Ghent, Belgium; ^3^Key Laboratory for Animal Disease-Resistance Nutrition of China, Institute of Animal Nutrition, Ministry of Education, Sichuan Agricultural University, Chengdu, China

**Keywords:** resistant starch, gut microbiota, SCFAs, bone mass, meat ducks

## Abstract

Gut microbiota interfered with using prebiotics may improve bone mass and alleviate the onset of bone problems. This study aimed to investigate the beneficial effect of resistant starch from raw potato starch (RPS) on bone health in meat ducks. Response to the dietary graded level of RPS supplementation, both tibia strength and ash were taken out linear and quadratic increase and positively correlated with increased propionate and butyrate levels in cecal content. Moreover, further outcomes of gut microbiota and micro-CT analysis showed the beneficial effect of RPS on bone mass might be associated with higher Firmicutes proportion and the production of short-chain fatty acids (SCFAs) in the cecum. Consistent with improving bone mass, SCFAs promoted phosphorus absorption, decreased the digestive tract pH, and enhanced intestinal integrity, which decreased the expression of pro-inflammatory genes in both gut and bone marrow, and consequently depressed osteoclastic bone resorption mediated by inflammatory cytokines. These findings highlight the importance of the “gut-bone” axis and provide new insight into the effect of prebiotics on bone health.

## Graphical Abstract

Potential mechanisms how supplementation of resistant starch improved the tibia mass of meat duck. Dietary supplementation of 12% raw potato starch modulated gut microbiota and concomitant short-chain fatty acids production, followed by inhibition of inflammation. Consequently, the bone resorption mediated by inflammatory cytokine was decreased, and eventually contributed to a net increase in bone mass.
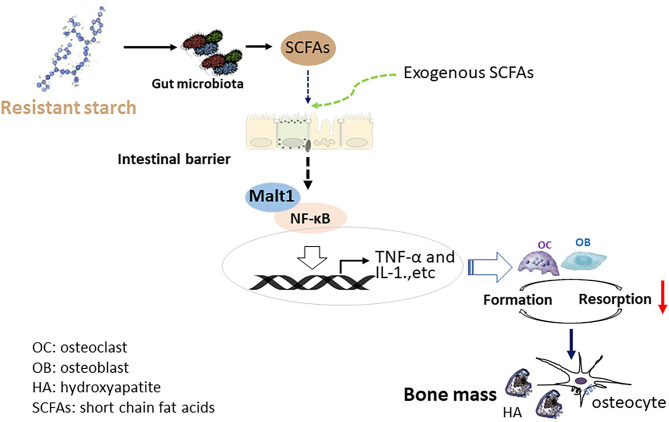

## Introduction

Gait problem in livestock is a welfare issue with a prevalence of about 14–21% in commercial meat ducks, which affects mainly modified behavior and pain (1). Birds with leg weakness are difficult to access to the food and water, and thus the gait problems might compromise growth performance. In response to this, a complex interplay between pathological and nutritional factors contributes to the etiopathogenesis of leg abnormalities (2). Of note, gut microbiome dysbiosis has been observed in both osteoporosis patients and experimentally ovariectomized rodent models (3). Manipulating microbiota (e.g., establishing germ-free, oral antibiotics, probiotics, etc.) could interfere with bone remodeling and bone quality through acting on the immune system, endocrine system, and calcium (Ca) absorption (4, 5). For instance, comparing germ-free mice with conventionally raised mice showed that the presence of microbiota led to lower trabecular and cortical bone mass (6), which companies with reduced number of osteoclasts and lower level of interleukin (IL)-6, receptor activator of nuclear factor-κ B ligand (RANKL), tumor necrosis factor-alpha (TNF-α), and CD4^+^T cells in bone (7, 8).

Diet and microbial metabolism were noticed to interact with the intestinal barrier and mucosal immune system to affect the immune responses and inflammation (9), which is possible to modify bone mass (4). Reports from our recent studies have demonstrated that impaired intestinal barrier and increased expression of inflammatory cytokines in ileum and bone marrow resulted in inferior tibia material properties and bone mass, and these features were further reversed by dietary 25-hydroxycholecalciferol treatment (10). The addition of propionate, a kind of short-chain fatty acid (SCFA), significantly relieved bone erosion and improved bone quality in the right knees from collagen-induced arthritis mice (11). In this context, as a subgroup of dietary fiber, resistant starch (RS) has been receiving great attention due to its health benefits on disease processes prevention, including arthritis (11) and bone loss (12). In addition to altering the composition of the gut microbiota, as evidenced by increasing the proportion of Bifidobacterium, Lactobacillus, and Firmicutes (13), RS could be also used as substrates by bacterial species that reside in the hindgut to generate important metabolites, including SCFAs (14). Ongoing research has provided important evidence for the positive role of RS on gut epithelial integrity and anti-inflammatory functions (9). Especially, we noticed that dietary supplements with 12% raw potato starch (RPS) as the resource of type 2 RS can thicken the mucosal layer, tighten the gut barrier, and decreased intestinal inflammation in meat ducks (14, 15). Pieces of evidence from the estrogen deficiency model have described significant remission in bone loss of mice given by 12% RS by regulating the intestinal microbiota and bone-marrow inflammation (12, 16, 17). Nevertheless, it is unclear how the bone characteristics and gut microbiota can be precisely modified by dietary graded RS supplementation in meat ducks. In this study, therefore, the responses of bone metabolism, inflammation, and the composition of intestinal microbiota to different levels of dietary RS were evaluated in meat ducks. Additionally, the key molecular factors and potential mechanism that determined how RS improved the bone quality was also explored.

## Materials and Methods

### Animals and Management

All experimental protocols were approved by the Animal Care and Use Committee of Henan Agricultural University and the animals were maintained in accordance with office guidelines for the care and use of laboratory animals. The 1-d-old Cherry Valley male ducks were purchased from Sichuan Mianying Breeding Duck Co. Ltd. and were housed (0.16 m^2^/bird) in a 12:12-h light-dark cycle room at 32°C for the 1–7 d, following which the temperature was decreased to 25°C at d 7 and 14. During the experiments, body weight, feed intake, and mortality were recorded, gain and feed conversion as the feed-to-gain ratio was calculated on a per-cage basis.

### Trial Design

Experiment 1. To investigate the dose-dependent effect of RS on bone characteristics and intestinal integrity, ducklings were fed the same basal diets with 0, 6, 12, or 24% RPS for 14 days. Each group had 6 replicate pens with 15 ducks per pen. Four isonitrogenous and isocaloric diets were formulated under a digestible amino acid basis to meet the nutrient requirements of duck suggested in the National Nutrition Council (18) and our previous studies (14) (Table 1). RPS was purchased from AVEBE Ltd. and contains 54.72% RS content (dry matter basis). RS was analyzed and confirmed proper preparation of experimental diets.

**Table 1:** Dietary formulation and composition (as fed basis).

**Item**	**Raw potato starch, % of diet weight**			
	**0**	**6**	**12**	**24**
**Ingredients, %**				
Corn	59.82	52.33	44.85	29.88
Raw potato starch	0	6.00	12.00	24.00
Soybean meal	33.21	34.57	35.91	38.61
Soybean oil	0.50	1.00	1.50	2.50
Calcium carbonate	1.10	1.07	1.04	0.98
Dicalcium phosphate	1.74	1.78	1.82	1.89
L-Lysine-HCL	0.11	0.09	0.07	0.04
DL-Methionine	0.16	0.16	0.16	0.17
Threonine	0.02	0.01	0.01	0.01
Bentonite	2.36	2.01	1.66	0.94
Sodium chloride	0.30	0.30	0.30	0.30
Choline chloride	0.15	0.15	0.15	0.15
Vitamin premix1	0.03	0.03	0.03	0.03
Mineral premix2	0.50	0.50	0.50	0.50
Total	100.0	100.0	100.0	100.0
**Calculated nutrient level, %**				
Apparent metabolism energy, MJ/kg	11.71	11.69	11.72	11.74
Crude protein	19.45	19.51	19.49	19.55
Calcium	0.91	0.89	0.92	0.9
Non-phytate phosphorus	0.43	0.41	0.42	0.39
Digestibility lysine	1.03	0.92	0.97	0.99
Digestibility methionine	0.41	0.43	0.42	0.42
**Analyzed nutrient level, %**				
Resistant starch	2.76	4.84	7.51	18.16

*^1^Provided per kilogram of diet: Cu (CuSO_4_ · 5H_2_O), 8 mg; Fe (FeSO_4_ · 7H_2_O), 80 mg; Zn (ZnSO_4_ · 7H_2_O), 90 mg; Mn (MnSO_4_ · H_2_O), 70 mg; Se (NaSeO_3_), 0.3 mg; I (KI), 0.4 mg*.

*^2^Provided per kilogram of diet: retinol, 2.06 mg; cholecalciferol, 0.04 mg; vitamin E, 30.01 mg; thiamine, 1 mg; riboflavin, 3.9 mg; pyridoxine, 3.375 mg; vitamin B_12_, 0.01 mg; calcium pantothenate, 8.85 mg; folate, 0.5 mg; biotin, 0.1 mg; niacin, 49.25 mg*.

Experiment 2. To define the relationship between SCFAs and bone turnover, birds at the day 1 were assigned to one of 3 trial groups (6 replicate pens; 15 ducks/pen) for 14 d as follows: (1) Birds were given 133.4 mM sodium chloride in drinking water and fed 0% RPS diets (salt-matched control, Ctrl); (2) Ducks consumed 133.4 mM sodium chloride in drinking water and 12% RPS diets; (3) Birds received SCFAs (67.5 mM sodium acetate, 38.8 mM sodium propionate, 22.8 mM sodium butyrate) in drinking water and fed 0% RPS diets. The proportion of SCFAs used in this study was based on previously reported (19) and kept the same ratio of acetate, propionate, and butyrate in the cecal content of meat duck fed 12% RPS diet in Experiment 1.

### Data Collection and Sampling

On day 14, ducks were weighed after 8 h feed withdrawal. Two birds per pen with body weight close to the pen average were selected. One was anesthetized for blood sampling through the jugular vein. Then, the animals were sacrificed by cervical dislocation, and the length and weight of the full ileum were measured, the pH of the ileal content was registered using an automated pH probe (pH-STAR, SFK-Technology, Denmark). Digesta samples of cecum were randomly divided into 2 parts. One portion was stored at −20°C for SCFAs determination and the other was kept in liquid N_2_ for 16S rDNA amplicon sequencing. In addition, the left tibia (the proximal end), bone marrow, duodenal and ileal mucosa were gained and stored at −80°C until further analysis. The right tibia was collected for tibia characteristics. Another was killed by cervical dislocation, and the left tibia (the proximal end) was gained for histological determination. Right tibias were removed for a Micro-CT scan after the removal of soft tissues.

### Determination of Gut Permeability

Fluorescein isothiocyanate-dextran (FITC-d) was used as an indicator of gut permeability in this study. In detail, 15 days old birds were received orally FITC-d (4.16 mg/kg body weight), and blood was harvested after 2 h. Serum fluorescence was analyzed at an excitation/emission wavelength of 485/530 nm using a Gemini XPS Microplate Reader (Molecular Devices, LLC. Sunnyvale, CA, USA). The content of serum FITC-d was calculated from the standard curves generated by the serial dilution of FITC-d.

### Analysis of Dietary RS

The RS content of diets was evaluated in accordance with AACC method 32-40.01 (20) using an assay kit (Megazyme International, Bray, Ireland). Briefly, non-resistant starch is solubilized and hydrolyzed to glucose by the combined action of pancreatic α-amylase and amyloglucosidase for 16 h at 37°C. The reaction is terminated by the addition of ethanol or industrial methylated spirits and RS is recovered as a pellet by centrifugation. RS in the pellet is dissolved in 2 mol/L KOH by vigorously stirring in an ice-water bath. This solution is neutralized with acetate buffer and the starch is quantitatively hydrolyzed to glucose with amyloglucosidase. Glucose is measured with glucose oxidase-peroxidase reagent, which is a measure of RS content.

### Measurement of the Serum Indicator

The concentrations of cytokines including TNF-α, IL-1β, and IL-10 were determined using available ELISA kits (Meimian Biotechnology Co., Ltd, Jiangsu, China). The concentration of secretory immunoglobulin A (IgA) in the ileum was assayed using Chicken ELISA Quantitation Kits (Bethyl Laboratories Inc., Montgomery, USA). Serum Ca and phosphorus (P) concentrations were measured with Biochemistry Analyzer (Yellow Springs Instrument Co. Inc., Yellow Springs, OH). The circulation of bone turnover markers including alkaline phosphatase (ALP) activity, procollagen type I N-terminal propeptide (P1NP) level, tartrate-resistant acid phosphatase (TRAP) activity, and C-terminal cross-linked telopeptide of type I collagen (CTx) content were assayed by ELISA assay (Nanjing Jiancheng Bioengineering Institute, Nanjing, China) following the manufacturer's instructions. All samples were tested in triplicate within each assay.

### Biochemical Indices of Mucosal Growth

Cell size and metabolic activity of ileal cells were estimated through measurements of mucosal protein, DNA, and RNA and the ratios between the 3 factors. The assay for these indices was performed using commercial kits (Nanjing Jiancheng Bioengineering Institute, Nanjing, China) according to the manufacturer's instructions.

### 16S RDNA Amplicon Sequencing of Cecal Microbiota

The total DNA in cecal content was extracted using a DNA stool mini kit (Qiagen, Valencia, United States) and was subjected to assess the integrity and size of DNA. Primers 515 F (5′-GTGYCAGCMGCCGCGGTAA-3′) and 806 R (5′-GGACTACHVGGGTWTCTAAT-3′) were used to amplify the hypervariable V3-V4 regions of the 16S rDNA gene. Then, the resulting PCR products were sequenced on an Illumina PE250 platform (BGI, Shenzhen, China). The obtained sequences were processed using FLASH (v1.2.11) and USEARCH (v7.0.1090) for alignment and clustering. All effective reads were clustered into operational taxonomic units (OTUs) with a similarity threshold of 97%. The representative sequence of each OTU was aligned against the Greengene database for taxonomy analysis. As for data analysis, alpha- and beta-diversity metrics were calculated using QIIME software and the R Vegan package. In detail, the alpha diversity was evaluated with MOTHUR at the genus level by calculating the Chao1 richness index (richness), reciprocal Simpson biodiversity index (diversity), and Simpson evenness index (evenness). Beta-diversity at the genus level was estimated by calculating Bray-Curtis dissimilarity and visualized with principal coordinates analysis (PCoA).

### Determination of SCFAs Concentrations in Cecal Content

According to previously described (14), the cecal content (approximately 0.5 g) was diluted with 2 ml of ultrapure water mixed, deposited for 30 min, and centrifuged at 3,000*g* for 15 min, followed by 1 ml supernatants were mixed with 0.2 ml ice-cold 25% (w/v) metaphosphoric acid solution and incubated at 4°C for 30 min. After centrifuging at 11,000*g* for 10 min, the SCFAs contents including acetate, propionate, and butyrate were separated and determined by gas chromatograph (Varian CP-3800, USA).

### Assessment of Tibia Microstructure Using Micro-CT

The microstructure of tibia metaphysis was measured using a GE Explore Locus Micro-CT (GE Healthcare, Piscataway, NJ, USA) at 90 kV with instrument settings optimized for calcified tissue visualization. The trabecular bone in the tibia metaphysis was performed starting from 9 mm below the surface of the condyles and extending 4 mm distally. Bone volume/total volume (BV/TV) and thickness (Tb.Th) of trabecular bone were calculated. The average thickness of the structures was measured using the thickness plugin from Bone J as our previous method following our recent methods (10).

### Detection of Tibia Mass and Strength

The breaking force test was determined with a 3-point bending test using the texture analyzer (TA. XT Plus; Stable Microsystems) with a constant 50 kg load cell. The tibia sample was placed on 2 vertically parallel supports. Loading proceeded at a constant rate (5 mm/min) until a fracture occurred, and the maximum load of the tibia was directly read from the peak value. Hereafter, fat-free weight was determined after extraction using ethyl ether for 48 h. Subsequently, the dry-defatted tibia was ashed in a muffle furnace at 550°C for 24 h and the ash was measured based on the percentage of dry-defatted weight.

### TRAP Staining

The fixed proximal end of the tibia was decalcified in ethylene diamine tetraacetic acid solution, embedded, and sliced. The decalcified section was de-paraffinized, rehydrated, and subsequently incubated for 1 h at 37°C with TRAP staining solution, according to the manufacturer's protocol (Sigma-Aldrich, Inc., St. Louis, MO, USA). Histopathological images were collected using a microscope with image analysis software (Nikon Corporation, Tokyo, Japan). The numbers of osteoclast/number of bone surface (N.Oc/BS) with multinucleated cells (≥3 nuclei) were identified as TRAP-positive and were determined in 5 consecutive microscopic fields. At least 5 serial vertical sections were evaluated for each animal per analysis. The slides were counted by 2 examiners, who were blinded to group status.

### Gene Expression Assays

RNA from ileal, duodenum, tibia, and bone marrow was extracted using Trizol (Invitrogen). After examining the integrity and concentration, the 200 ng of total RNA was reversely transcribed into cDNA. Quantitative real-time PCR was performed on the ABI 7900HT detection system (Applied Biosystems, CA, USA). Relative gene expression was quantified by normalizing the expression of β*-actin* and glyceraldehyde-3-phosphate dehydrogenase (*GAPDH*). The primer sequences for the target genes were designed using Primer 3 (Table 2).

**Table 2:** The primers for quantitative real-time PCR.

**Gene**	**Gene ID**	**Primer**	**Sequence (5^**′**^-3^**′**^)**	**Size (bp)**
*OPG*	XM_005017709.3	Reverse	gcctaactggctgaacttgc	106
		Forward	gaaggtctgctcttgcgaac	
*RANKL*	XM_021276016.1	Reverse	gccttttgcccatctcatta	100
		Forward	taagtttgcctggcctttgt	
*ZO-1*	XM_013104939.1	Reverse	tacgcctgtgaagaatgcag	86
		Forward	ggagtggtggtgtttgcttt	
*Occludin*	XM_013109403.1	Reverse	caggatgtggcagaggaatacaa	160
		Forward	ccttgtcgtagtcgctcaccat	
*Claudin 1*	XM_013108556.1	Reverse	tcatggtatggcaacagagtgg	179
		Forward	cgggtgggtggataggaagt	
*Malt1*	XM_027446455.2	Reverse	ccatggaaaccgtacttgct	118
		Forward	ttgtgcaggggattggtaat	
*NF-κB*	XM_027455993.1	Reverse	gagcgttttcaagaggttgc	123
		Forward	agggatcttctcctgccatt	
*TNF-α*	EU375296.1	Reverse	agatgggaagggaatgaacc	51
		Forward	gttggcataggctgtcctgt	
*IL-1β*	DQ393268.1	Reverse	gcatcaagggctacaagctc	131
		Forward	caggcggtagaagatgaagc	
*IL-6*	AB191038.1	Reverse	atctggcaacgacgataagg	87
		Forward	ttgtgaggagggatttctgg	
*IL-17*	EU366165.1	Reverse	atgcctgacccaaaaagatg	145
		Forward	gtggtcctcatcgatcctgt	
*IL-18*	XM_027444356.2	Reverse	ctgatgacgatgagctggaa	120
		Forward	caaaagctgccatgttcaga	
*GPR41*	KJ523111.1	Reverse	actgacgtcctcctcctcaa	160
		Forward	tggtgaggtagatgctggtg	
*GPR43*	KJ523110.1	Reverse	agcagctgagctttgtcctc	129
		Forward	gtggaatattaggccgagca	
*NaPi-IIb*	NM_204474.3	Reverse	gctccagcacttcttcatcc	95
		Forward	aatgtttgcccccataatga	
*Calbindin 1*	XM_027452451.2	Reverse	cagggtgtcaaaatgtgtgc	109
		Forward	gatccttcagcagtgcatca	
*β-actin*	NM_001310408.1	Reverse	ccagccatctttcttgggta	105
		Forward	gtgttggcgtacaggtcctt	
*GAPDH*	XM_005016745.3	Reverse	tttttaaccgtggctccttg	94
		Forward	actgggcatggaagaacatc	

*OPG, osteoprotegerin; RANKL, receptor activator of nuclear Factor-κ B ligand; ZO-1, zonula occludens; Malt1, mucosa-associated lymphoid tissue lymphoma translocation protein 1; NF-κB, nuclear factor kappa B; TNF-α, tumor necrosis factor alpha; IL, interleukin; GPR, G protein-coupled receptor; NaPi-IIb, sodium-dependent phosphorus transport protein IIb; GAPDH, glyceraldehyde-3-phosphate dehydrogenase*.

### Statistical Analysis

Statistical analyses were performed using SAS statistical software (version 9.2, SAS Institute, Cary, NC). After checking for normal distribution and equal variance using the Shapiro–Wilk and Levene's tests, respectively. The ANOVA followed by Tukey's *post-hoc* test was conducted to examine statistical significance. Polynomial contrasts of growth performance, ileal physiochemical parameter, SCFAs level, and bone characterizing the response to dietary RPS level were examined using linear and quadratic regression. Spearman's rank correlation coefficient test was used for assessing the association between the SCFAs content, tibia strength, and ash. Broken-line regression analysis was used to estimate the recommended level of dietary RPS supplementation using the non-linear regression (NLIN) procedure of SAS (SAS Institute). Data were expressed as mean and standard deviation (SD). The *p*-values less than 0.05 were considered significant.

## Results

### Effects of Dietary Graded RPS on Growth Performance and Intestinal Physiochemical Environment

As shown in Table 3, dietary graded levels of RPS supplementation quadratically affected the body weight on day 14 (*p* = 0.069) and the feed conversion ratio expressed as feed to gain ratio during 1–14 days (*p* < 0.001). Notable changes in feed intake and mortality during all experimental periods were not observed among all groups. Therewith, the intestinal physiochemical environment was inferred from the ileal length, weight, relative weight, and pH, as well as cecal SCFAs (Table 4). The relative weight of ileum remarkably increased as supplemented dietary RPS level (*p* = 0.023). However, the administration of dietary RPS failed to change the length and absolute weight of ileum. The pH of ileal content showed a decreased trend in the 6, 12, and 24% RPS groups when compared with the 0% RPS diet. Regarding cecal SCFAs level, dietary RPS supplementation had no effect on acetate contents in the cecal digesta, whereas the contents of both propionate and butyrate in the cecum were taken out linear and quadratically increased response to the dietary graded concentration of RPS. When compared to the 0% RPS group, the diet with 12 and 24% RPS significantly increased the level of propionate and butyrate in the cecum (*p* < 0.05), implying that diet with 12 and 24% RPS promoted propionate and butyrate production in the cecum and tended to decrease the pH of the digestive tract.

**Table 3:** Effect of dietary raw potato starch (RPS) supplementation on growth performance for meat ducks.

**Item**	**RPS, % of diet weight**				***P*** **-value**		
	**0**	**6**	**12**	**24**	**ANOVA**	**Linear**	**Quadratic**
Body weight at 14 d, g/bird	552.51 ± 27.94	592.1 ± 16.46	596.89 ± 20.86	578.86 ± 28.35	0.056	0.236	0.069
Feed intake during 1–14 d, g/bird	654.81 ± 3.24	655.78 ± 2.89	656.18 ± 2.94	654.57 ± 3.22	0.773	0.801	0.565
Feed intake: gain during 1–14 d, g/g	1.19 ± 0.06	1.11 ± 0.04	1.10 ± 0.04	1.22 ± 0.06	0.063	0.229	<0.001
Mortality during 1–14 d, %	3.33 ± 5.58	2.22 ± 3.44	1.11 ± 2.72	2.22 ± 3.44	0.813	0.635	0.626

**Table 4:** Effect of dietary raw potato starch (RPS) supplementation on the ileal physiochemical environment and cecal short chain fat acids (SCFAs) level in 14 day-old meat ducks.

**Item**	**RPS, % of diet weight**				***P*** **-value**		
	**0**	**6**	**12**	**24**	**ANOVA**	**Linear**	**Quadratic**
Ileum							
Length, cm	62.43 ± 1.54	65.34 ± 1.52	65.30 ± 3.08	64.71 ± 3.44	0.191	0.265	0.118
Weight, g	7.81 ± 0.47	7.92 ± 0.33	8.27 ± 0.47	8.38 ± 0.63	0.165	0.031	0.091
Relative weight, % body weight	1.43 ± 0.13^b^	1.35 ± 0.09^b^	1.39 ± 0.06^ab^	1.56 ± 0.17^a^	0.025	0.029	0.009
pH	6.82 ± 0.23	6.58 ± 0.35	6.43 ± 0.31	6.34 ± 0.35	0.068	0.012	0.026
Cecal SCFAs, μmol/g							
Acetate	55.55 ± 6.21	57.25 ± 7.28	58.44 ± 5.82	60.4 ± 5.72	0.602	0.166	0.386
Propionate	27.23 ± 3.65^b^	30.6 ± 3.16^ab^	33.55 ± 2.67^a^	32.77 ± 3.73^a^	0.017	0.013	0.006
Butyrate	13.80 ± 2.83^b^	16.53 ± 4.18^ab^	19.69 ± 2.52^a^	19.65 ± 2.35^a^	<0.001	0.003	0.029

a,b *Mean values with different letters are significantly different by one-way analysis of variance followed by Tukey's post-hoc test (p < 0.05)*.

### Tibia Growth, Strength, and Ash Response to Dietary RPS Supplementation

**Figure 1:**
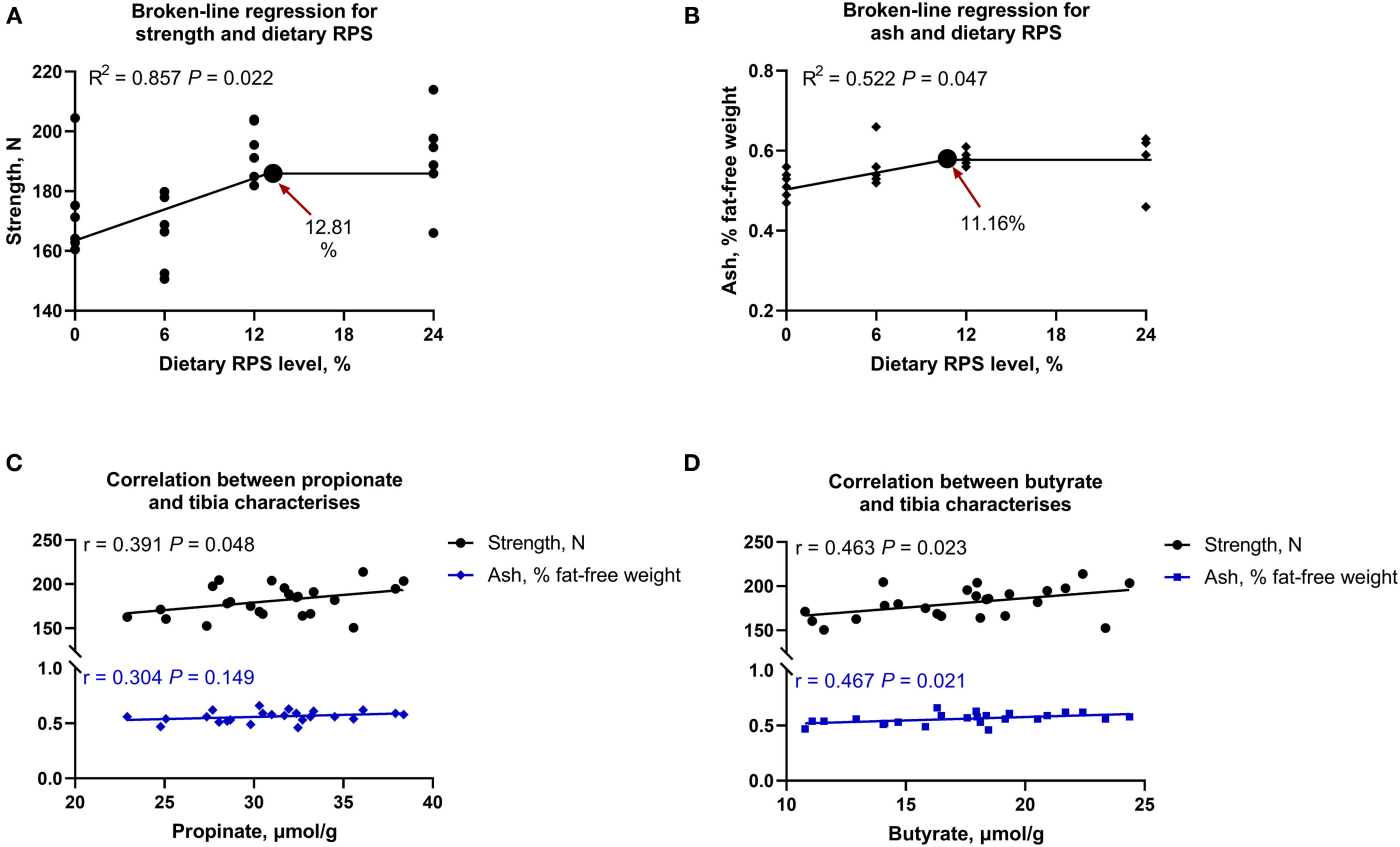
The recommended level of dietary raw potato starch (RPS) based on broken-line analysis of **(A)** tibia strength and **(B)** ash content, as well as Pearson's correlation **(C)** between tibia strength and ash and propionate level in cecal content, and **(D)** between tibia strength and ash and butyrate level in cecal chyme.

Tibia growth was assessed through bone length, diameter, and fat-free weight, and there was no significant difference in the aforementioned parameters among all groups (*p* > 0.05; Table 5). As dietary supplemental RPS levels increased, tibia strength and ash content increased linearly and quadratically (Table 5). Furthermore, to understand the connections between SCFAs change and bone characteristics, the Broken-line regression analysis between dietary graded RPS levels and tibia mass was performed and showed that both tibia strength and ash were increased with dietary RPS intervention, where the recommended level of dietary RPS was 12.81 and 11.16% based on tibia strength and tibia ash, respectively (Figures 1A,B). Meanwhile, Pearson's correlation analysis among significantly changed propionate, butyrate, tibia strength, and ash was also conducted. As shown in Figures 1C,D, the tibia strength was positively correlated with an increasing level of propionate (*r* = 0.391, *p* = 0.048) and butyrate (*r* = 0.463, *p* = 0.023). In addition, the tibia ash content was also positively associated with the levels of butyrate (*r* = 0.467, *p* = 0.021), but not propionate (*p* > 0.05). Collectively, the dietary supplementation with 12% RPS could improve tibia quality in 14 days-old meat ducks, which might be associated with SCFAs production.

**Table 5:** Effect of dietary RPS supplementation on the tibia characterizes of 14 day-old meat ducks.

**Item**	**RPS, % of diet weight**				***P*** **-value**		
	**0**	**6**	**12**	**24**	**ANOVA**	**Linear**	**Quadratic**
Tibia growth							
Length, mm	72.63 ± 2.46	72.62 ± 2.35	73.08 ± 2.58	73.01 ± 2.81	0.983	0.640	0.734
Diameter, mm	5.51 ± 0.27	5.57 ± 0.35	5.65 ± 0.20	5.59 ± 0.33	0.887	0.745	0.941
Fat-free weight, g	1.50 ± 0.16	1.57 ± 0.35	1.67 ± 0.17	1.76 ± 0.27	0.305	0.056	0.159
Tibia quality							
Strength, *N*	173.08 ± 16.37^b^	166.01 ± 12.34^b^	193.52 ± 9.27^a^	191.17 ± 15.75^a^	0.015	0.012	0.040
Ash, g/g fat-free weight	0.52 ± 0.03	0.56 ± 0.05	0.58 ± 0.02	0.59 ± 0.06	0.053	0.021	0.020

a,b *Mean values with different letters are significantly different by one-way analysis of variance followed by Tukey's post-hoc test (p < 0.05)*.

### Dietary 12% RPS and Drinking SCFAs Improve Tibia Quality Associated With Inhibited Bone Resorption and Increased P Absorption

**Figure 2:**
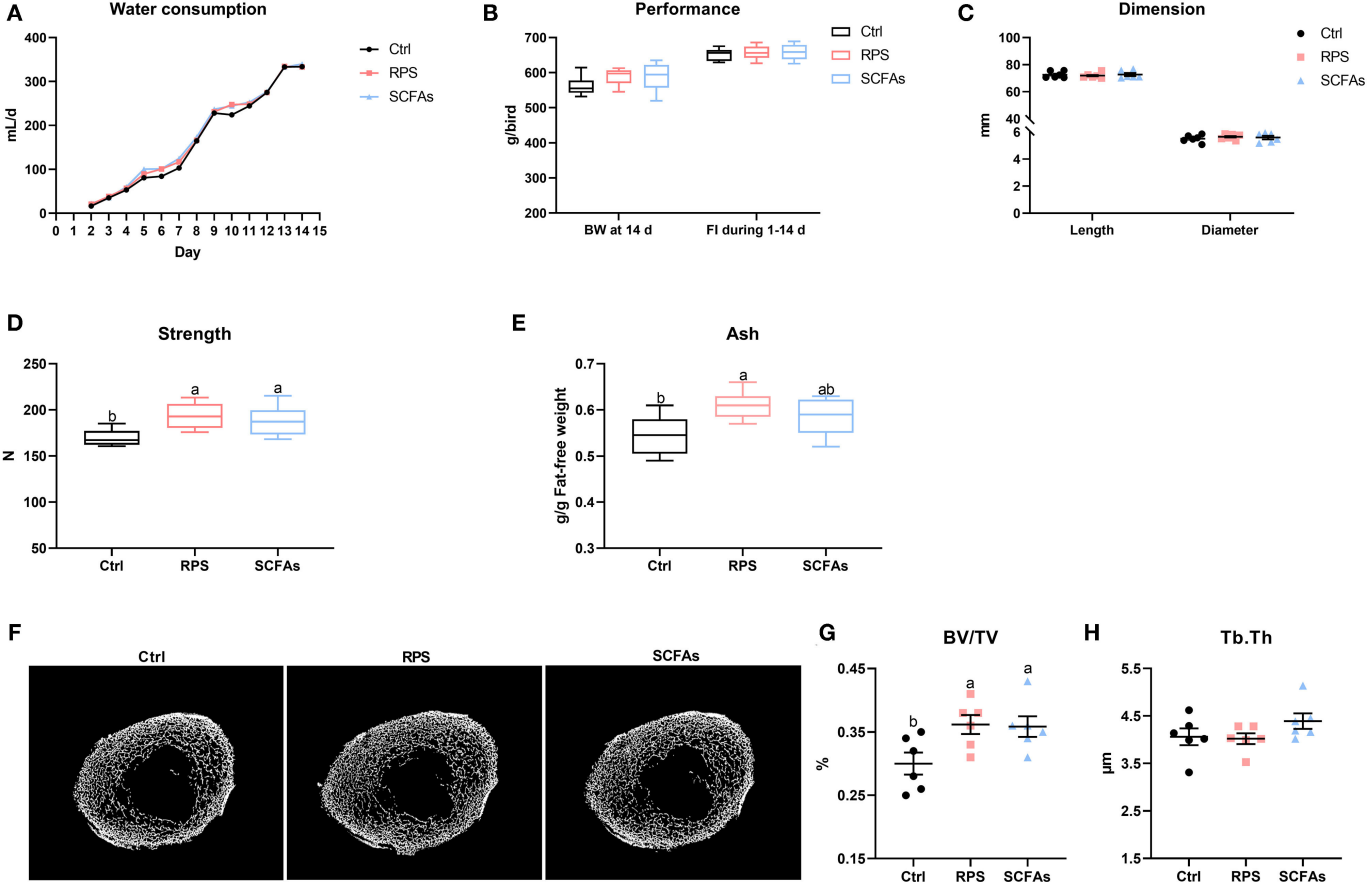
Dietary RPS and drinking short chain fatty acids (SCFAs) addition improve tibia mass in meat ducks. **(A)** Water consumption, **(B)** performance including body weight at 14 day and feed intake during 1–14 days, and **(C)** tibia length and diameter were determined. The bone quality was indicated by **(D)** tibia strength and **(E)** ash. **(F)** Representative micro CT images and the quantification of **(G)** bone volume/total volume (BV/TV) and **(H)** trabecular thickness (Tb.Th) of the proximal tibia. Data are expressed as mean and standard deviation. ^a,b^ Mean values with different letters are significantly different by one-way analysis of variance followed by Tukey's *post-hoc* test (*p* < 0.05).

To further determine whether SCFAs produced by RPS-associated gut bacteria are the pivotal factors contributing to the improvement of tibia mass in meat ducks, the exogenous addition of SCFAs in drinking water was used and showed that the experimental treatments did not apparently change the daily water consumption, body weight on the day 14, feed intake during 1–14 days, and tibia length and diameter at the day 14 (Figures 2A–C). As expected, as compared with the Ctrl group, both dietary 12% RPS and drinking SCFAs notably increased tibia strength and BV/TV of the proximal tibia (*p* < 0.05). Similarly, dietary 12% RPS supplementation and drinking SCFAs treatment increased tibia ash to varying degrees, although Tb.Th trabecular among these groups did not differ (Figures 2D–G), suggesting that dietary 12% RPS addition improved the bone mass of meat ducks might be involved in SCFAs production.

**Figure 3:**
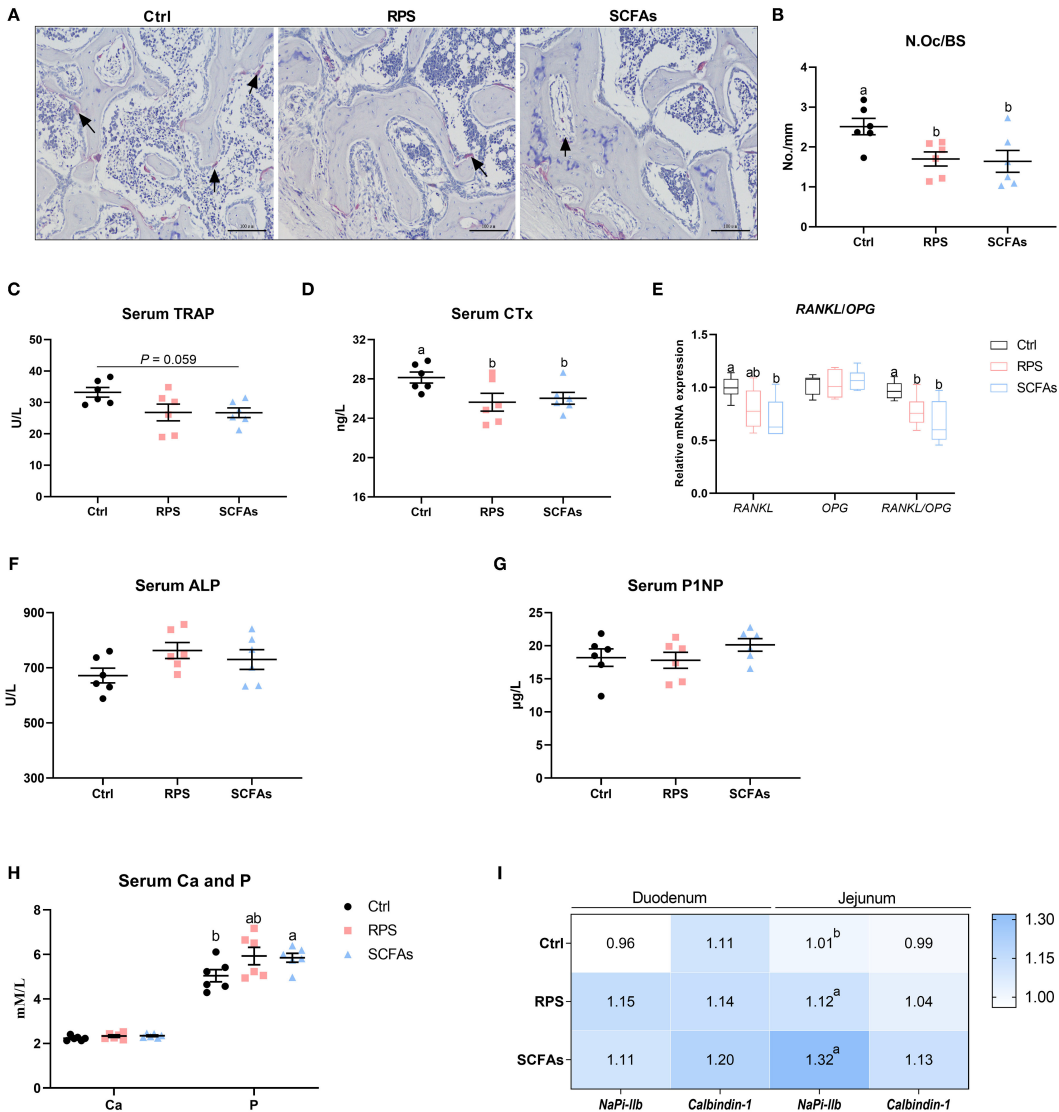
Dietary RPS and drinking SCFAs administration suppressed osteoclastic bone resorption and induced phosphorus (P) absorption in meat ducks. **(A)** Tartrate resistant acid phosphatase (TRAP) staining of tibial sections. Bar = 100 μm. **(B)** The number of osteoclast (N.Oc/BS) in proximal tibias was determined by histomorphometry. Circulating **(C)** TRAP activity and **(D)** C-terminal cross-linked telopeptide of type I collagen (CTx) concentrations. **(E)** Real-time (RT)-PCR analysis for mRNA expression of receptor activator for nuclear factor-κB ligand (*RANKL*) and osteoprogerin (*OPG*) in the proximal end, and the ratio of *RANKL*/*OPG* was calculated. Serum bone formation including **(F)** procollagen type I N-terminal propeptide (P1NP) and **(G)** alkaline phosphatase (ALP) level, as well as **(H)** serum calcium (Ca) and phosphorus (P) concentration were evaluated. **(I)** The mRNA abundance of RT-PCR analysis for mRNA expression of sodium-dependent phosphorus transport protein II (*NaPi-IIb*) and *calbindin-1* in duodenum and ileum. Data are expressed as mean and SD. ^a,b^ Mean values with different letters are significantly different by one-way analysis of variance followed by Tukey's *post-hoc* test (*p* < 0.05).

Effects of dietary 12% RPS and drinking SCFAs administration on bone resorption were assessed histologically and biochemically. TRAP-positive cells were distinctly observed in Ctrl birds (Figure 3A). N.Oc/BS was reduced by approximately 32 and 35% by the dietary 12% RPS and drinking SCFAs intervention, respectively (Figure 3B). Circulating bone resorption markers, TRAP and CTx, were decreased by dietary 12% RPS and drinking SCFAs treatment (Figures 3C,D). We then examined the mRNA expression of osteoclastogenesis-related factors in bone, the experimental treatments did not significantly alter the mRNA level of osteoprogerin (*OPG*), whereas both dietary 12% RPS and drinking SCFAs addition decreased the expression of *RANKL* mRNA to varying degrees (*p* < 0.05), thereby decreased the *RANKL*/*OPG* ratio (Figure 3E). Additionally, bone formation was also assessed by serum biochemical parameters. The serum ALP activity was numerically increased by dietary 12% RPS and drinking SCFAs (*p* > 0.05; Figure 3F). No appreciable changes in the P1NP concentrations were observed among the 3 groups (Figure 3G). Moreover, the effects of drinking SCFAs and dietary 12% RPS on Ca and P absorption were shown in Figure 3H. Serum P concentration tends to increase due to dietary 12% RPS treatment and was notably elevated by drinking SCFAs, whereas serum Ca concentration was unaffected (*p* > 0.05). No difference was observed in terms of the mRNA expressions of *calbindin-1* and sodium-dependent phosphorus transport protein IIb (*NaPi-IIb*) in the duodenum. However, dietary 12% RPS and drinking SCFAs treatment resulted in a remarkable increase in the transcription of *NaPi-IIb* in jejunum when compared with the Ctrl group (*p* < 0.05; Figure 3I). Taken together, these results suggest that feeding the 12% RPS diet improves tibia quality associated with inhibited bone resorption and increased P absorption.

### Dietary 12% RPS and Drinking SCFAs Treatment Increase the Abundance of Firmicutes and Promote Concomitant SCFAs Production

**Figure 4:**
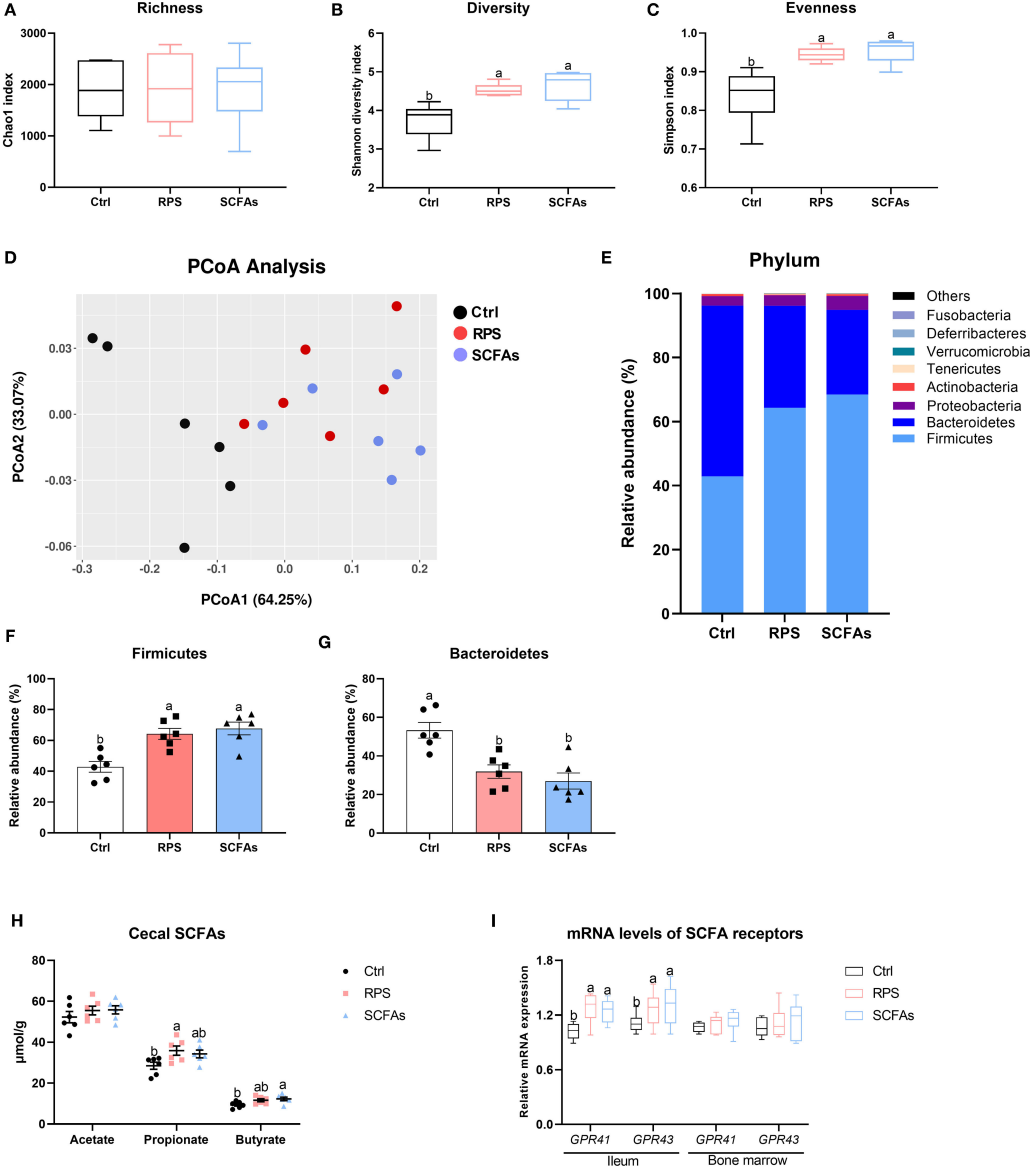
Dietary RPS and drinking SCFAs change the microbiota composition and promote concomitant SCFAs production in the cecum of meat ducks. **(A–C)** Chao1, Shannon, and Simpson indexes were used to assess diversity and evenness of gut microbiota. **(D)** Principle coordinate analyses (PCoA) of beta diversity based on Bray-Curtis dissimilarities of bacterial operational taxonomic units. **(E)** Relative phylum level abundance of gut bacteria. The proportion of **(F)** Firmicutes and **(G)** Bacteroidetes. **(H)** SCFAs production and **(I)** SCFA receptor gene expression including G protein-coupled receptors 41 (*GPR41*) and *GPR43* in the ileum and bone marrow. Data are expressed as mean and standard deviation. ^a,b^ Mean values with different letters are significantly different by one-way analysis of variance followed by Tukey's *post-hoc* test (*p* < 0.05).

Concerning the effects of the RPS and exogenous SCFAs administration on the caecal microbiome, the alpha diversity was significantly increased by experimental treatment, indicated by higher Shannon and Simpson indexes in both RPS- and SCFAs-treated ducks than it in the Ctrl birds, although with comparable Chao index among three groups (Figures 4A–C). Diversity metrics that utilize species richness and evenness (Bray-Curtis) showed a statistically apparent separation between the dietary 12% RPS and Ctrl groups, and the profile of group dietary 12% RPS was partly overlapped with that of the drinking SCFAs group (Figure 4D). In addition, remarkable differences in microbial composition were also demonstrated in the phylum-level distribution patterns (Figure 4E). Compared with the Ctrl group, dietary 12% RPS and drinking SCFAs intervention notably promoted the abundance of Firmicutes and meanwhile decreased the content of Bacteroidetes (Figures 4F,G). Besides, the outcomes from quantifying SCFAs in cecal content showed that the administration of dietary 12% RPS and drinking SCFAs failed to change the proportion of acetate, whereas they increased the concentration of propionate and butyrate at different levels (Figure 4H). Reflecting the mRNA expressions of SCFA receptors including *GPR41* and *GPR43* in the ileum and bone marrow, which were significantly upregulated by dietary 12% RPS and drinking SCFAs treatment in the ileum but not bone marrow as compared with the Ctrl groups (Figure 4H).

### Effect on Intestinal Barrier and Biochemical Indices of Mucosal Growth

**Figure 5:**
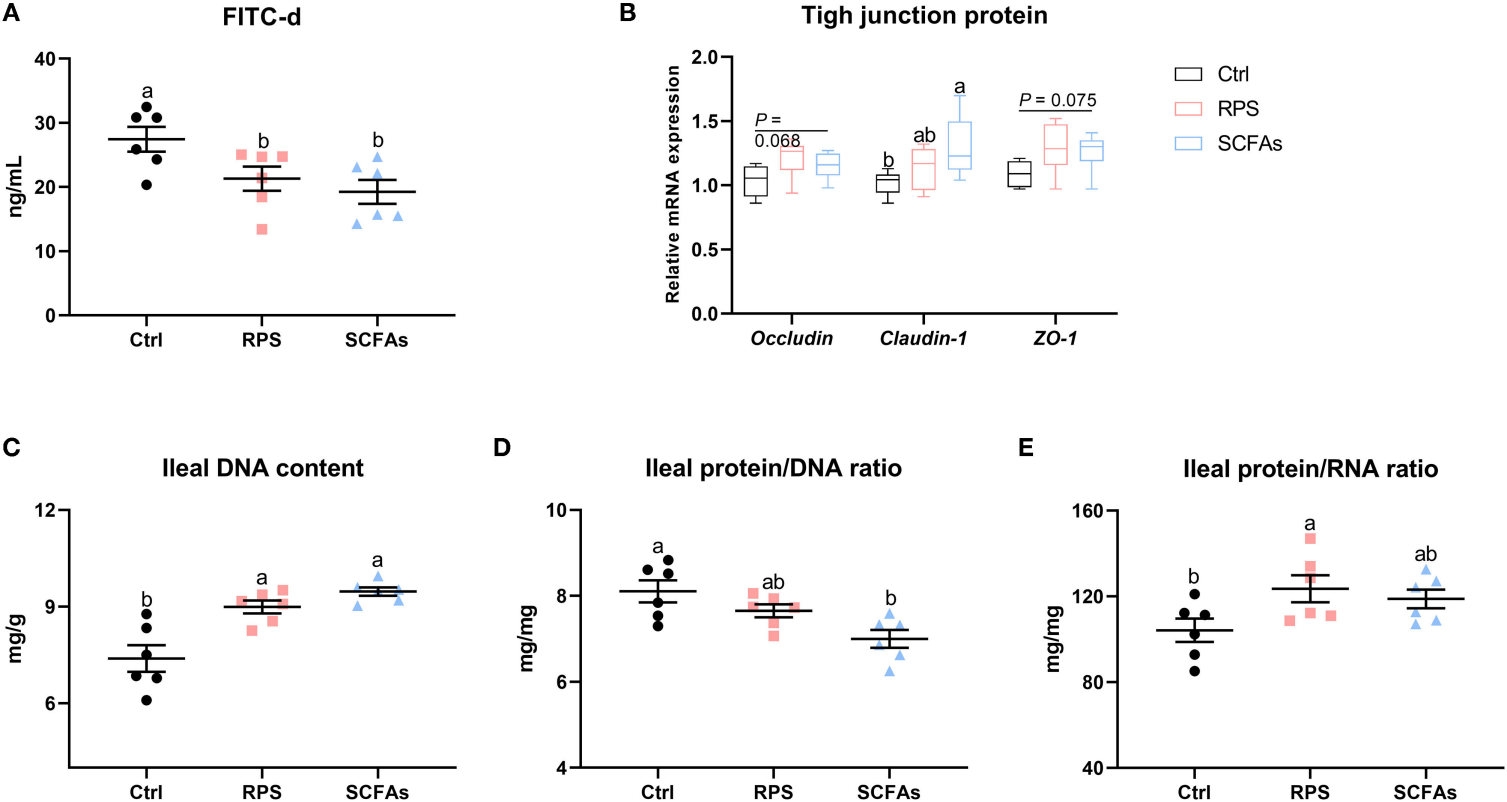
Dietary RPS and drinking SCFAs enhance intestinal barrier and biochemical indices of mucosal growth. Intestinal integrity was assessed by **(A)** direct measurement using fluorescein isothiocyanate dextran (FITC-d) and **(B)** the mRNA level of tight junction proteins including *occluding, caudin-1*, and zona occludens-1 (*ZO-1*). Biochemical indices of mucosal growth including **(C)** DNA content, **(D)** protein/RNA ratio, and **(E)** protein/RNA ratio of the ileal mucosal homogenate were assessed. Data are expressed as mean and standard deviation. ^a,b^ Mean values with different letters are significantly different by one-way analysis of variance followed by Tukey's *post-hoc* test (*p* < 0.05).

A direct comparison of the concentration of FITC-d suggests that supplementation of dietary 12% RPS and drinking SCFAs significantly decreased intestinal permeability, evidenced by lower serum FITC-d concentration than it in the Ctrl group (Figure 5A). Further examination of tight junction proteins (TJPs) confirmed that the expressions of *occludin*, zonula occludens-1 (*ZO-1*), and *claudin-1* were numerically upregulated by the administration of dietary 12% RPS and drinking SCFAs as compared to Ctrl birds (Figure 5B). As far as mucosal growth of ileum is concerned, when compared with the Ctrl birds, higher DNA content and protein/RNA ratio, as well as a lower protein/RNA ratio of the ileal mucosal homogenate were observed in ducks that received a 12% RPS diet and drinking SCFAs addition (Figures 5C–E). Together, these data suggest that supplementation with a 12% RPS diet and drinking SCFAs enhanced intestinal barrier function.

### Dietary 12% RPS and Drinking SCFAs Addition Alleviated Inflammatory Reaction in Both Gut and Bone Marrow

**Figure 6:**
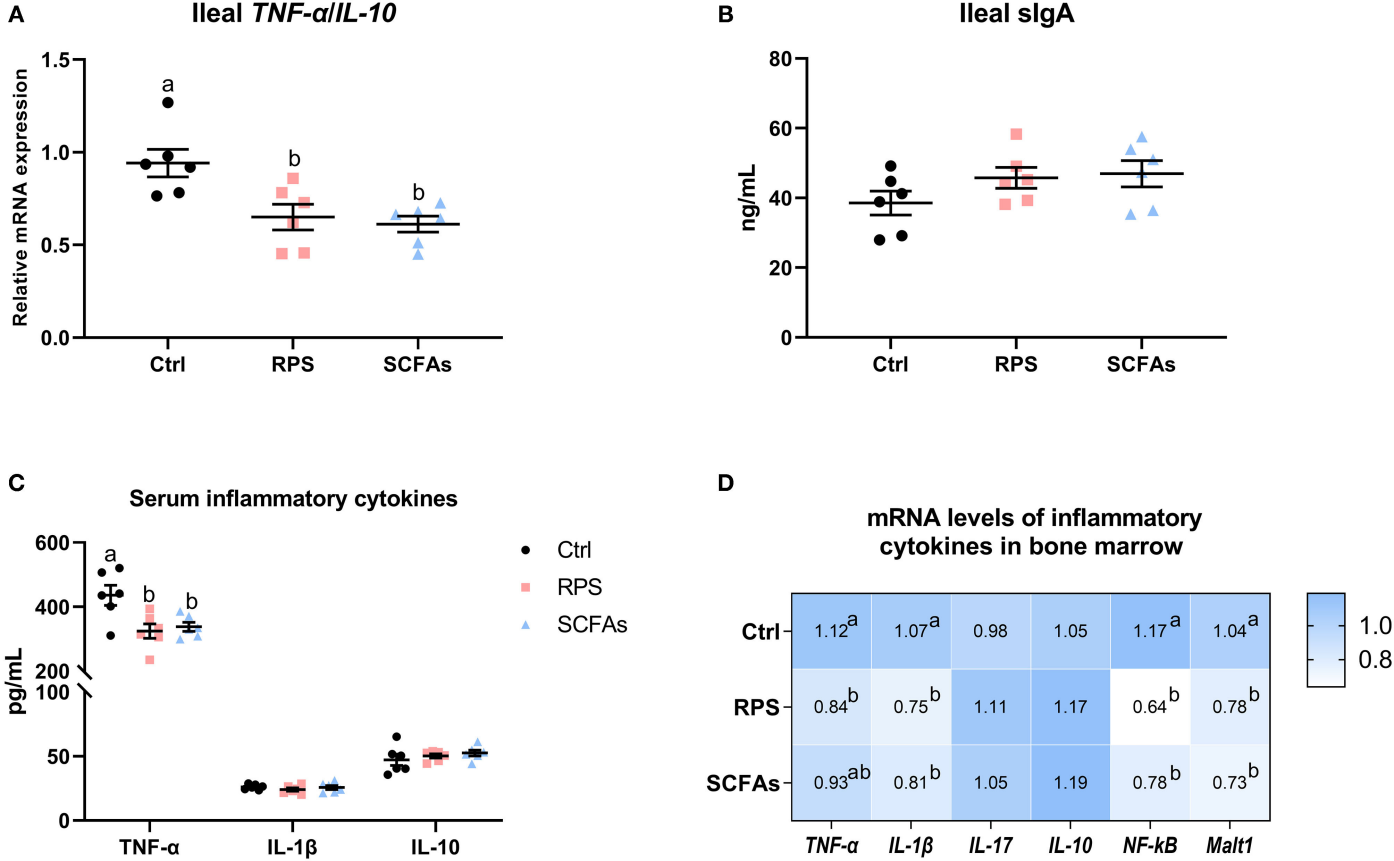
Dietary RPS and drinking SCFAs are associated with alleviative inflammatory reactions. **(A)** The ratio of tumor necrosis factor alpha (*TNF-*α) and interleukin (*IL*)-*10*, and **(B)** the content of secretory immunoglobulin A (IgA) in the ileum. **(C)** The levels of TNF-α, IL-1β, and IL-10 in serum from different groups. **(D)** The mRNA expression of inflammatory cytokines in bone marrow including *TNF-*α, *IL-1*β, *IL-17, IL-10*, nuclear factor-κB (*NF-*κ*B*), and mucosa-associated lymphoid tissue lymphoma translocation protein 1 (*Malt1*). Data are expressed as mean and standard deviation. ^a,b^ Mean values with different letters are significantly different by one-way analysis of variance followed by Tukey's *post-hoc* test (*p* < 0.05).

Analyses of ileum pro- and anti-inflammatory cytokine expression revealed that the manipulation of dietary 12% RPS and drinking SCFAs notably decrease the *TNF-*α/*IL-10* ratio in the ileum (*p* < 0.05; Figure 6A), whereas these treatments did not obvious change the ileal sIgA level as compared with Ctrl group (Figure 6B). With comparable *IL-1*β and *IL-10* content, the ducks that were given a 12% RPS diet and drinking SCFAs exhibited lower serum TNF-α content relative to Ctrl birds (Figure 6C). In the bone marrow, the higher mRNA levels of *TNF-*α, *IL-1*β, nuclear factor-κB (*NF-*κ*B*), and mucosa-associated lymphoid tissue lymphoma translocation protein 1 (*Malt1*) in Ctrl birds were decreased by the 12% RPS diet and drinking SCFAs addition. In contrast, the mRNA expressions of *IL-17* and *IL-10* were similar among all groups (Figure 6D). Thus, these data suggest that manipulation of dietary 12% RPS and drinking SCFAs was accompanied by a notably decreased inflammation in the gut and bone marrow.

## Discussion

Increasing numbers of studies demonstrate a role for the gut microbiota, as well as its composition in the regulation of bone health (21). Here, using 16S rDNA sequencing and micro-CT techniques, this research characterized the gut microbial alteration in response to dietary RS supplementation from RPS and explored a potential connection between prebiotic intervention and bone mass. Our finding showed that the intake of dietary 12% RPS improves tibia quality in meat ducks by modulating gut microbiota and promotes concomitant propionate and butyrate production.

Previous reports in mice have evidenced the protective effects of dietary RS against bone loss induced by estrogen deficiency (12) and collagen-induced arthritis (11). It is, therefore, plausible that appropriate dietary RS supplementation might alleviate the gait problems for meat ducks, in which the prevalence of gait abnormalities is about 14–21% in commercial duck populations (1). With this aim, the different levels of dietary RPS were assessed and showed that both tibia strength and tibia ash were increased with dietary RPS intervention, where the recommended level of dietary RPS was 12.81 and 11.16% for optimizing tibia strength and ash based on broken-line regression analysis, respectively. One of the potential mechanisms underlying the beneficial role on bone quality may attribute to the intestinal microbiota and concomitant SCFAs production (4, 11). In fact, as a subgroup of dietary fiber, RS can be used as substrates by bacterial species to produce some fermented products such as SCFAs (14). Several studies have characterized the capacity of RS to induce alterations in the composition of the gut microbiota, including the increases in Bifidobacterium, Lactobacillus, and Bacteroides (13). More importantly, it has been suggested that SCFAs generated from prebiotics could regulate the intestinal and systemic inflammation reaction, thereby alleviating bone metabolism (22), which was further supported by our data. In the present study, the dietary graded level of RPS supplementation linearly and quadratically increased the contents of propionate and butyrate in the cecum digest and positively correlated with tibia strength and ash to varying degrees, respectively.

How do alterations in gut microbiota and concomitant SCFAs production induced by dietary RS promote bone mass? It was established that dysbiosis has been associated with bone loss in patients and experimentally ovariectomized rodent models (3) and manipulating intestinal microbiota *via* oral antibiotics and production germ-free mice could apparently affect bone quality in rodent models (6, 23). In this study, along with the improvement of tibia strength, ash, and BV/TV of proximal bone, the dietary 12% RPS treatment significantly affected the microbial composition demonstrated by the higher proportion of Firmicutes and the lower the abundance of Bacteroidetes, which multiplied proven by our team previous studies (14). In this regard, published literature pointed out that reduced Firmicutes/Bacteroidetes ratio was linked with declined bone loss of mice (24). An increase in the proportion of Firmicutes and a decrease in Bacteroidetes due to dietary RS intervention was critical correlations with the aggravation in disease manifestations in collagen-induced arthritis mice (11). These discrepancies might interpret into that the regulation of gut microbiota on bone is not only attributed to the ratio of Firmicutes and Bacteroidetes, but also the generation of fermentation, especially SCFAs. Indeed, Firmicutes bacteria produce high amounts of butyrate and propionate, such as *Faecalibacterium prausnitzii, Roseburia sp*., and *Eubacterium rectale*, whereas Bacteroidetes bacteria produce high levels of acetate and propionate (25, 26). Accordingly, in the current study, the elevated content of Firmicutes resulted in a higher concentration of propionate and butyrate in cecal chyme that is consistent with our previous observations (14). Therefore, it is possible that the SCFAs but not the Firmicutes/Bacteroidetes ratio might act as the primary mediators to promote tibia property in meat ducks.

To further determine whether SCFAs produced by dietary RS-associated gut bacteria are the pivotal factors contributing to the improvement of tibia quality in meat ducks, the direct addition of SCFAs into drinking water was used in the present study. Analogous to dietary 12% RPS-treated birds, SCFAs-treated ducks had higher tibia strength, ash, and BV/TV compared to Ctrl ducks. The addition of propionate was also observed to significantly restore bone erosion and bone loss in the right knees from collagen-induced arthritis mice (11). Considering the role of SCFAs in promoting gut development through stimulating the secretion of glucagon-like peptide (GLP)-1 and GLP-2 from enteroendocrine L cells (27, 28), enhancing barrier function to suppress bone resorption mediated by inflammation probably is a critical contributor for dietary 12% RPS diets promoting bone strength and mass. As expected, in response to the increased levels of butyrate and propionate, SCFAs receptors *GPR41* and *GPR43* in ileum were notably upregulated by dietary 12% RPS like the effects of drinking SCFAs in this study. Subsequently, the biochemical indices of mucosal growth were estimated through measurements of mucosal protein, DNA, and RNA and the ratios between the three factors. In detail, changes in DNA reflect variations in cell population because the DNA content of diploid cells remains unchanged after cell formation. The ratio of protein/DNA is an indicator of cell size, while the ratio between protein and RNA measures the rate of protein synthesis (29). In the present study, higher DNA content and protein/RNA ratio, and lower protein/RNA ratio of the ileal mucosal homogenate implies that the administration with a 12% RPS diet and drinking SCFAs promoted the proliferation and activity of intestinal cells. As a result, we noticed that the addition of dietary 12% RPS enhanced the intestinal barrier of meat ducks, evidenced by lower serum FITC-d concentration and upregulated mRNA level of TJPs.

Alterations of intestinal permeability have been associated with bone loss in diseases such as inflammatory bowel disease (30). Under the condition of impairing gut integrity, bacteria and their factors probably translocate across the intestinal barrier to induce systemic inflammatory responses (31). Our recent studies have also demonstrated that impaired intestinal barrier and increased expression of inflammatory cytokines in ileum and bone marrow resulted in inferior tibia material properties and bone mass, which were further reversed by dietary 25-hydroxycholecalciferol treatment (10). In the present study, using the ileum TNF-α/IL-10 ratio as a marker of the balance between a pro- vs. anti-inflammatory state found that dietary 12% PRS and drinking SCFAs addition decreased the ileal inflammation, which accompanied with lower serum TNF-α level. Of note, dietary 12% RPS treatment also downregulated the mRNA expression of *Malt1, NF-*κ*B, TNF-*α, and *IL-1*β in the bone marrow. The production of proinflammatory cytokines is governed by NF-κB signaling that dependent on the involvement of Malt1. Malt1 plays a key role in innate and adaptive immunity as a scaffold to activate the NF-κB signaling or exert a proteolytic activity to fine-tune gene expression in myeloid cells and non-immune cells (32). Inhibiting Malt1 could prevent the activation of NF-κB signaling thus reducing IL-1β and IL-18 secretion in mice (33). Therefore, these results suggest that dietary 12% RPS supplementation decreased the secretion of the pro-inflammatory cytokine which might be due to the inactivation in Malt1/NF-κB signaling. In particular, receptors to SCFAs are highly expressed on innate immune cells, especially neutrophils, and function as an “anti-inflammatory chemoattractant receptor” (34), implying that the alteration in microbiota composition that allows for SCFAs production function may modulate neutrophil recruitment during inflammatory responses. The linking of inflammation and bone metabolism has been well defined (35). For instance, mice with TNF-α induced arthritis and possessed increased osteoclast precursors in serum, and subsequently was reversed by anti-TNF-α therapy and correlated with systemically increased TNF-α concentrations (36). The germ-free mice with higher trabecular and cortical bone mass were also characterized by a reduced number of osteoclasts and lower level of IL-6, RANKL, TNF-α, and CD4+ T cells in bone marrow when compared with conventionally raised mice (7, 8). Meanwhile, in the current study, while increasing bone ash and strength, we also found that dietary 12% RPS inclusion reduced N.Oc/BS, circulating TRAP, and CTx level, as well as the *RANKL* mRNA level and the *RANKL*/*OPG* ratio. Of note, RANKL binds to RANK that is expressed on the surface of osteoclast to induce osteoclast differentiation, whereas OPG acts as a decoy receptor by blocking the interaction of RANKL with its functional receptor RANK (37). The downregulated *RANKL* expression and thereby declined *RANK*/*OPG* ratio in dietary 12% RPS fed-birds probably explained the reduced osteoclast number in the tibia. In accordance with previous research saying that dietary RS could attenuate the bone loss in ovariectomized mice (12, 16, 17), this study also further confirmed that dietary 12% RPS supplementation inhibited the bone resorption and improved tibia mass and strength in meat ducks.

Another potential mechanism by which dietary 12% RPS enhanced bone quality might be related to P absorption. Increased SCFAs concentration produced by RPS fermentations tends to acidify the intestinal environment and facilitate mineral retention such as Ca and total P. In the present study, Ca content in serum did not influence by experimental treatments, whereas serum P concentration was increased due to dietary 12% RPS and drinking SCFAs administration, which was probably attributed to the upregulated transcription of *NaPi-IIb*, a regulator of P transport in the intestine (38). Studies in broiler chicken also confirmed that declining the pH of the gastrointestinal tract produced better ileal nutrient digestibility and improved P absorption (39). Analogous to SCFAs, the organic acid supplementation together with the developing desirable gut microflora was deemed to contribute to mineral retention and bone mineralization through increased digestibility and availability of nutrients (40). Accordingly, remarkably higher Ca and P blood concentrations were observed in chicks fed a diet supplemented with organic acids such as acetic, citric, and lactic acid, and further demonstrated that the beneficial effects of organic acids were caused by the lowering of gut pH and the increase in the absorption of these macro-elements (41). By contrast, manipulation with a 3% inclusion of citric acid in diet did not change blood Ca and P concentrations in broiler chickens (42). These variations or comparable serum Ca content in this study might be regarded to feed ingredients or/and the endocrine regulation for maintaining the homeostasis of Ca and P (43).

There are three new questions that need to be further elucidated in the following research. The first is the effect of dietary RS and age on bone formation. Our previous works concluded that the tibia of meat duck exhibits rapid bone growth and mineralization from 1 to 35 days (44). Theoretically, therefore, the supplementation of dietary RPS and its fermentation by gut microbiota could affect the rate of bone formation, which is inconsistent with the current study saying that serum bone formation markers such as ALP and P1NP abundance failed to be changed by dietary 12% RPS or drinking SCFAs treatment. Further studies are necessary to draw a precise conclusion on the mechanisms of observed interactions between the prebiotics and bone formation. The second is the direct role of SCFAs on bone-related cells. Undifferentiated GPR41 and GPR43 transcription of bone marrow in this study suggested that SCFAs may not directly act on the bone to interact with bone turnover. Previous studies also deemed that a direct effect of SCFAs on bone resorption *in vivo* is unlikely to interfere with the differentiation of bone cells (21). Nevertheless, more accurate knowledge of the interactions between SCFAs and bone-related cells is still required. The third is the methodology; there are only methods available to measure RNA but not protein in this study, especially for some proteins related to the Malt1/NF-κB inflammasome. Therefore, we admit the possibility that some of our conclusion may include overestimation or underestimation of roles of dietary RPS in inflammation and subsequent bone resorption. Further experiments using Western blotting would be essential to elaborate on this possibility.

## Conclusion

In the present study, dietary 12% RPS supplementation beneficially affected tibia mass in 14 day-old meat ducks through modifying gut microbiota and bone resorption. Results of gut microbiota showed that dietary 12% RPS intervention notably changed the relevant abundance of bacteria, which was associated with higher production of cecal propionate and butyrate. In addition to regulating the physiochemical environment to stimulate P absorption *via* decreasing the digestive tract pH, SCFAs enhanced intestinal integrity and depressed osteoclastic bone resorption mediated by inflammatory cytokines, and consequently improved tibial quality. These findings are of preliminary nature that highlights the importance of the “gut-bone” axis in bone health, provides ideas for further investigations into the precise mechanisms, and gives a new perspective for nutritional application of dietary fermentable fibbers-related products.

## Data Availability Statement

The datasets presented in this study can be found in online repositories. The names of the repository/repositories and accession number(s) can be found below: https://www.ncbi.nlm.nih.gov/genbank/, SRR7370079.

## Ethics Statement

The animal study was reviewed and approved by Institutional Animal Care and Use Committee at the Henan Agricultural University.

## Author Contributions

HZ, QZ, and WC: experimental design. QZ, WC, and JM: supervision and reviewing. HZ, SQ, and YZ: conducted the experimental and sample collection. HZ, SQ, XZ, PD, YZ, and YH: data analysis and wrote-original draft. All authors reviewed and approved the final manuscript.

## Funding

This study was supported by grants from the National Natural Science Foundation of China (31772622), the National Natural Science Foundation of China (32072748), and a Doctoral Fellowship from Henan Agricultural University (0501182).

## Conflict of Interest

The authors declare that the research was conducted in the absence of any commercial or financial relationships that could be construed as a potential conflict of interest. The reviewer (SB) declared a shared affiliation with the authors to the handling editor at the time of review.

## Publisher's Note

All claims expressed in this article are solely those of the authors and do not necessarily represent those of their affiliated organizations, or those of the publisher, the editors and the reviewers. Any product that may be evaluated in this article, or claim that may be made by its manufacturer, is not guaranteed or endorsed by the publisher.
